# Circulating miRNome profiling data in Behçet's syndrome

**DOI:** 10.1016/j.dib.2021.107435

**Published:** 2021-09-28

**Authors:** Giacomo Bagni, Giacomo Emmi, Elena Lastraioli, Francesca Di Patti, Elena Silvestri, Angela Guerriero, Serena Pillozzi, Elena Niccolai, Amedeo Amedei, Lorenzo Emmi, Domenico Prisco, Annarosa Arcangeli

**Affiliations:** a Department of Experimental and Clinical Medicine, University of Florence, Florence, Italy; b SOD Interdisciplinary Internal Medicine-Behçet Center and Lupus Clinic–Azienda Ospedaliero Universitaria Careggi, Florence, Italy; c Department of Physics, University of Florence, Sesto Fiorentino, Florence, Italy; d Center for the Study of Complex Dynamics (CSDC), Sesto Fiorentino, Florence, Italy; e Department of Neuroscience, Psychology, Drug Research and Child Health, University of Florence, Florence, Italy

**Keywords:** microRNA, Circulating miRNAs, Behçet, Microarray, Biomarker

## Abstract

We conducted a screening analysis to assess the presence of a characteristic extracellular circulating microRNAs (ci-miRNAs) profile in Behçet's syndrome (BS).

Total RNA was extracted from platelets-free plasma (PFP) samples obtained from 16 BS patients and 18 healthy controls. Ci-miRNAs profiling was conducted by using dedicated Agilent microarray hybridization and data extraction technology. Statistical analysis of data extracted from microarray scanning revealed the deregulation of 36 ci-miRNAs, which turned out be differentially expressed between BS patients and healthy controls. Detailed experimental methods and data analysis were described here.

The raw and normalized microarray data were deposited into Gene Expression Omnibus (GEO) under accession number GSE145191.

## Specifications Table

**Table:** 

Subject	Biology
Specific subject area	Circulating microRNA in Behçet's syndrome
Type of data	Table
How data were acquired	Data were acquired by using the dedicated Agilent miRNA Microarray Technology. Instruments: Agilent microarray G2565A Scanner, Agilent G2545A hybridization oven, Agilent Human miRNA 8 × 15k Microarray kit (v3.0), miRNA Complete Labeling and Hyb Kit, Agilent Feature Extraction (AFE) (v.9.1), AgiMicroRna R script (available on Bioconductor repository) (v3.12).
Data format	Raw Analyzed Filtered
Parameters for data collection	miRNA Microarray profiling performed on total RNA extracted from the plasma of 16 Behçet's syndrome [1] patients and 18 healthy controls using Trizol LS.
Description of data collection	Data obtained by performing miRNA Agilent Microarray hybridization experiments, followed by data extraction and analysis using available dedivcated scripts for data pre-processing and expression analysis. Expression data were compared between patients and healthy controls.
Data source location	Institution: Department of Experimental and Clinical medicine, University of Florence City: Florence Country: Italy Latitude and longitude for collected samples/data: 43.9027681, 11.2463084
Data accessibility	Repository name: Gene Expression Omnibus (GEO) database. Data identification number: GSE145191 Direct URL to data: https://www.ncbi.nlm.nih.gov/geo/query/acc.cgi?acc=GSE145191

## Value of the Data

• The provided data represent the extracellular ci-miRNAs profile of patients affected by Behcet's syndrome, a rare systemic vasculitis.
• The data provide basis for the prediction of deregulated ci-miRNAs association with pathogenic pathways in Behçet's syndrome via *in silico* target analysis.
• The data could potentially provide guidance for the discovery of non-invasive biomarker candidates for BS diagnosis by validation in a larger and independent cohort.

## 1 Data Description

### 1.1 Patients and healthy controls

Sixteen BS patients and 18 HC were included in the present study as the microarray screening cohort.

No statistically significant difference in both age and sex between the two groups was detected.

Independently from the different baseline clinical features and from the active manifestations at the enrollment, our cohort well represented the different phenotypes of BS (namely the mucocutaneous and articular, the ocular and neurological and the vascular manfestations) [1].

Demographic features of patients and controls included the microarray screening cohort are reported in Table 1.

**Table 1:** Demographics of the patients and controls included in the microarray screening cohort.

	BS	HC
*n* (subjects observed)	16	18
Sex *(n, %)*		
Male	9 (56.3)	6 (33.3)
Female	7 (43.8)	12 (66.6)
Age *Mean (IQR; range)*	42.5 (8.5; 26-52)	36.3 (12; 28-40)

Data presented as mean with interquartile range or number (*n*) and relative percentage when applicable. No statistically significant differences were found between groups for mean age and sex ratio evaluated by Student's T test and Chi-square test respectively. BS= Behçet's syndrome patients; HC= heathy controls; IQR=Interquartile Range.

### 1.2 Deregulated ci-miRNAs identification

RNA samples were subjected to miRNA profiling using the dedicated Agilent microarray technology. The statistical analysis of the whole miRNome data revealed the presence of 36 DE (*p* < 0.05; -1 > log_2_ FC > 1) ci-miRNAs between BS patients and HC, 17 down-regulated and 19 up-regulated (listed in Table 2). The maximum up-regulation and down-regulation fold change values were, respectively, 2.45 and -2.19. The identified profile mainly comprised human sequences (indicated by the “hsa” prefix) with only 7 entities being of viral origin.

**Table 2:** DE miRNAs identified by microarray analysis in the screening phase.

miRNA ID	MIMATID	Sequence	FC	P	FDR
**hsa-miR-653-5p**	MIMAT0003328	guguugaaacaaucucuacug	24.544	0.0005	0.18047
**hsa-miR-224-5p**	MIMAT0000281	ucaagucacuagugguuccguuuag	20.148	0.0027	0.18047
**hsa-miR-206**	MIMAT0000462	uggaauguaaggaagugugugg	20.056	0.0037	0.18047
**hsa-miR-558**	MIMAT0003222	ugagcugcuguaccaaaau	19.013	0.0072	0.23381
**hsa-miR-573**	MIMAT0003238	cugaagugauguguaacugaucag	18.562	0.0222	0.34292
**hsa-miR-593**	MIMAT0003261	aggcaccagccaggcauugcucagc	17.143	0.0433	0.48152
**hsa-miR-425-3p**	MIMAT0001343	aucgggaaugucguguccgccc	16.772	0.0133	0.30151
**hsa-miR-189**	MIMAT0000079	ugccuacugagcugauaucagu	15.837	0.0144	0.30151
hsa-miR-525*	MIMAT0002839	gaaggcgcuucccuuuagagcg	14.999	0.0152	0.21726
**hsa-miR-200°**	MIMAT0000682	uaacacugucugguaacgaugu	14.419	0.0055	0.34292
ebv-miR-BHRF1-2*	MIMAT0000996	aaauucuguugcagcagauagc	14.385	0.0229	0.30112
**hsa-miR-601**	MIMAT0003269	uggucuaggauuguuggaggag	14.341	0.0100	0.21726
**hsa-miR-100**	MIMAT0000098	aacccguagauccgaacuugug	14.236	0.0054	0.35404
**hsa-miR-608**	MIMAT0003276	aggggugguguugggacagcuccgu	14.009	0.0245	0.45757
**hsa-miR-569**	MIMAT0003234	aguuaaugaauccuggaaagu	13.756	0.0399	0.34292
*ebv-miR-BART1-5p*	MIMAT0000999	ucuuaguggaagugacgugcugug	13.104	0.0217	0.44399
*ebv-miR-BART14-3p*	MIMAT0003426	uaaaugcugcaguaguagggau	11.894	0.0376	0.30791
**hsa-miR-376a**	MIMAT0000729	aucauagaggaaaauccacgu	11.229	0.0166	0.41419
**hsa-miR-627**	MIMAT0003296	gugagucucuaagaaaagagga	10.759	0.0329	0.30151
**hsa-miR-302b**	MIMAT0000715	uaagugcuuccauguuuuaguag	-11.783	0.0449	0.48519
**hsa-miR-98**	MIMAT0000096	ugagguaguaaguuguauuguu	-12.594	0.0329	0.41419
**hsa-miR-520e**	MIMAT0002825	aaagugcuuccuuuuugaggg	-14.332	0.0287	0.39922
*ebv-miR-BART6-3p*	MIMAT0003415	cggggaucggacuagccuuaga	-16.170	0.0069	0.23381
**hsa-miR-340**	MIMAT0004692	uuauaaagcaaugagacugauu	-16.206	0.0363	0.44212
**hsa-miR-566**	MIMAT0003230	gggcgccugugaucccaac	-16.358	0.0155	0.30151
*kshv-miR-K12-7*	MIMAT0002187	ugaucccauguugcuggcgc	-16.632	0.0222	0.34292
**hsa-miR-423**	MIMAT0001340	agcucggucugaggccccucagu	-17.271	0.0330	0.41419
*kshv-miR-K12-9*	MIMAT0002185	cuggguauacgcagcugcguaa	-18.083	0.0187	0.33113
hsa-miR-519e*	MIMAT0002828	uucuccaaaagggagcacuuuc	-18.331	0.0130	0.30151
**hsa-miR-432**	MIMAT0002814	ucuuggaguaggucauugggugg	-18.483	0.0144	0.30151
**hsa-miR-31**	MIMAT0000089	aggcaagaugcuggcauagcu	-19.111	0.0111	0.30151
*kshv-miR-K12-1*	MIMAT0002182	auuacaggaaacuggguguaagc	-20.371	0.0026	0.18047
**hsa-miR-411-5p**	MIMAT0003329	uaguagaccguauagcguacg	-21.903	0.0013	0.18047
**hsa-miR-187-3p**	MIMAT0000262	ucgugucuuguguugcagccgg	-21.927	0.0037	0.18047
**hsa-miR-27a-3p**	MIMAT0000084	uucacaguggcuaaguuccgc	-22.675	0.0034	0.18047
**hsa-miR-600**	MIMAT0003268	acuuacagacaagagccuugcuc	-23.197	0.0033	0.18047

Human miRNAs (indicated by “hsa” prefix) are in bold while viral ones are in italic, P values were calculated by two-tailed Student T test. MIMATID=unique mature miRNA accession number. FC=normalized expression fold change values in log2 scale. P=Limma (Linear models for Microarray Data) differential expression t-test p-value. *n*=34 (16 BS patients *vs* 18 HC). FDR= False Discovery Rate false discovery rate (determined according to the Benjamini-Hoechberg's method).

Complete microarray data (both as raw and processed datasets) are available in the NCBI's Gene Expression Omnibus (GEO) database under series accession number GSE145191, in accordance with the MIAME guidelines. For each sample, the overall miRNA profile is available both as raw and normalized mean fluorescence intensity values (contained respectively in the GEO Supplementary file and Sample table) associated to each microarray probe.

Samples characteristics and GEO data description are provided in Table 3.

**Table 3:** GEO microarray data description.

GSM number	Sample name	Group	Age	Sex
GSM4308223	B_PFP_2	BS	26	M
GSM4308224	B_PFP_6	BS	42	M
GSM4308225	B_PFP_7	BS	29	M
GSM4308226	B_PFP_8	BS	49	M
GSM4308227	B_PFP_9	BS	46	M
GSM4308228	B_PFP_10	BS	35	M
GSM4308229	B_PFP_11	BS	47	F
GSM4308230	B_PFP_14	BS	49	F
GSM4308231	B_PFP_15	BS	38	F
GSM4308232	B_PFP_16	BS	47	M
GSM4308233	B_PFP_17	BS	44	F
GSM4308234	B_PFP_18	BS	38	F
GSM4308235	B_PFP_19	BS	43	F
GSM4308236	B_PFP_21	BS	48	F
GSM4308237	B_PFP_22	BS	52	M
GSM4308238	B_PFP_23	BS	40	M
GSM4308239	C_PFP_24	HC	40	M
GSM4308240	C_PFP_25	HC	38	M
GSM4308241	C_PFP_26	HC	35	F
GSM4308242	C_PFP_27	HC	28	M
GSM4308243	C_PFP_28	HC	40	F
GSM4308244	C_PFP_30	HC	39	F
GSM4308245	C_PFP_31	HC	38	F
GSM4308246	C_PFP_32	HC	33	F
GSM4308247	C_PFP_33	HC	30	F
GSM4308248	C_PFP_34	HC	28	M
GSM4308249	C_PFP_35	HC	35	M
GSM4308250	C_PFP_36	HC	37	F
GSM4308251	C_PFP_38	HC	38	F
GSM4308252	C_PFP_39	HC	39	M
GSM4308253	C_PFP_40	HC	37	F
GSM4308254	C_PFP_41	HC	39	F
GSM4308255	C_PFP_42	HC	40	F
GSM4308256	C_PFP_43	HC	40	F

BS= Behçet's syndrome patients; HC= healthy controls; F= female; M= male; GSM= GEO sample accession number.

## 2 Experimental Design, Materials and Methods

### 2.1 Patients and healthy controls

Sixteen BS patients, fulfilling the International Criteria for Behçet's Disease (ICBD) [2], were recruited at the Florence Behçet Center (Azienda Ospedaliero-Universitaria Careggi), while 18 healthy controls (HC) were enrolled as blood donors at the Transfusional Medicine Centre of Azienda Ospedaliero-Universitaria Careggi.

All subjects were free from any laboratory or clinical sign of malignancies, infections or other immune-mediated diseases at blood sampling.

### 2.2 Sample collection and handling

Eight ml of peripheral blood were collected from each subject in BD Vacutainer K2 EDTA tubes (BD, Franklin Lakes, NJ, USA) by standard venipuncture. Among all commonly used anticoagulants, EDTA was selected due to its reported minimal effect on the circulating microRNA (ci-miRNA) profile during sample preparation step, as well as for the absence of interference with downstream applications [3]. Platelets Free Plasma (PFP) was obtained from peripheral blood samples by a double centrifugation protocol (1500 g for 15 min at room temperature followed by careful supernatant collection and centrifugation at 13,000 g for 3 min to eliminate platelets). Supernatants were carefully collected (making sure not to disturb the pellet) and finally aliquoted into fresh 1.5 ml RNAse-free tubes and stored at -80°C until use. The PFP plasma preparation was selected considering that the accurate and reliable measurement of extracellular ci-miRNAs is dependent on the removal of residual platelets prior to freezing plasma sample. Any sample showing clots or signs of hemolysis (red/pink plasma discoloration against a white background, indicative of severe hemolyzed samples) by visual inspection was excluded from the analysis, considering the well-known ability of this features to significantly alter plasmatic miRNA quantification [4]. All blood samples were sent to our laboratory and processed within 2 h from collection, since several evidences reported how peripheral blood cells, including erythrocytes, can contribute to extracellular miRNAs found in plasma and serum following longer term storage [5].

### 2.3 RNA extraction and quality control

Total RNA was extracted starting from a volume of 0, 25 ml of plasma. Trizol-LS reagent was used, following the manufacturer's protocol. RNA concentration and purity were assessed using the NanoDrop ND-2000 spectrophotometer (Thermo Fisher Scientific Inc., Waltham, Massachusetts, USA). Qualitative sample analysis was performed using the Agilent 2100 Bioanalyzer system and Small RNA Assay Chips (5067–1548, Agilent Technologies, Santa Clara, California, USA). Only Samples showing acceptable concentration and quality were included in the analysis.

### 2.4 Microarray hybridization

Circulating miRNome profiling was performed using the Agilent Human miRNA 8 × 15k Microarray kit v3.0 and the miRNA Complete Labeling and Hyb Kit (Agilent Technologies, Santa Clara, CA, USA) following the manufacturer instructions.

Briefly, For each sample, a total amount of 100 ng of total RNA was dephosphorylated, 3′ end-labelled with Cy3-pCp and dried. Cy3-labeled RNA in hybridization buffer was hybridized overnight (20 h, 55°C) to Agilent Human microRNA Microarray Chips using the recommended hybridization chamber and oven. Following hybridization, the microarrays were washed with the Agilent Gene Expression Wash Buffer 1 for 5 min at room temperature followed by a second wash with preheated Agilent Gene Expression Wash Buffer 2 (37°C) for 5 min before scanning.

### 2.5 Data extraction, pre-processing and differential expression analysis

Following microarray chips scanning procedure expression data were extracted from the TIFF images obtained from the G2565 Agilent's Microarray Scanning System (using a scan protocol with a resolution of 3 µm and a dynamic range of 20 bits) using the integrated Agilent Feature Extraction (AFE) v9.1 software (Agilent Technologies, Santa Clara, CA, USA). In order to perform data pre-processing and differential expression analysis, the processed information contained in the data source file were the following: gTotalGeneSignal, gMeanSignal (also contains background information), gIsGeneDetected, ControlType, ProbeName, and GeneName. Files were then processed using the R library AgiMicroRna v3.12 package, available on Bioconductor repository (https://bioconductor.org/packages/release/bioc/html/AgiMicroRna.html) [6].

This package allows for raw data pre-processing using a variant of the robust multi-array average (RMA) algorithm that has been specifically implemented for Agilent miRNA microarrays.

This pre-processing method has been shown to have better precision than the one recommended by Agilent [5].

Firstly, probe summarization was based on the median expression value for the replicated probes.

Median background values were then subtracted from the median expression values obtained from the AFE software. After obtaining the normalized total gene signal, probe signals were filtered based on the quality flags that AFE algorithm attaches to each feature. The quantile method was then applied in order to perform inter-array normalization (“normalizeBetweenArrays” function) in order to compensate for systematic technical differences between probe affinity and arrays.

After background correction, statistical significance of differences between study groups (BS patients *vs* HC) was assessed comparing mean microarray fluorescence intensity values using the Limma (Linear models for Microarray Data) differential expression t-test. A 2.0 times fold expression cutoff was applied to minimize the effect of probe background signal.

Finally, log2 transformation was used to obtain standardized expression values.

Deregulated (DE) miRNAs were identified from the microarray analysis on the basis of a *p* < 0.05 by two-tailed t-test and log_2_-scale fold change > 1 or < -1.

To account for discrepancies in miRNA nomenclature due to the different miRBase releases, array features names were converted to MIMATIDs (unique mature miRNA accession number) using the mapping file available in miRBase (http://www.mirbase.org).

### 2.6 Statistical analysis

Unless otherwise stated, categorical variables were presented with counts and proportions, while continuous ones as the mean ± standard error of the mean (SEM) or median with IQR (interquartile range). All statistical tests were two tailed with a significance level of 0.05.

## Ethics Statement

This study was conducted according to the Helsinki Declaration and approved by the local ethical committee of Azienda Ospedaliero-Universitaria Careggi, Florence, Italy (protocol number CE 13972). A signed written informed consent was obtained from each participant enrolled in the study.

## CRediT authorship contribution statement

**Giacomo Bagni:** Investigation, Formal analysis, Methodology, Software, Data curation, Validation, Visualization, Writing – original draft, Writing – review & editing. **Giacomo Emmi:** Conceptualization, Supervision, Project administration, Funding acquisition, Writing – original draft, Writing – review & editing. **Elena Lastraioli:** Methodology, Data curation, Visualization, Investigation. **Francesca Di Patti:** Investigation, Formal analysis, Methodology, Software, Data curation, Validation, Writing – original draft, Writing – review & editing. **Elena Silvestri:** Investigation, Formal analysis, Methodology, Software, Data curation, Validation. **Angela Guerriero:** Investigation, Formal analysis, Methodology, Software, Data curation, Validation, Visualization, Writing – original draft. **Serena Pillozzi:** . **Elena Niccolai:** Methodology, Data curation, Visualization, Investigation. **Amedeo Amedei:** Conceptualization, Supervision, Project administration, Funding acquisition. **Lorenzo Emmi:** Conceptualization, Supervision, Project administration, Funding acquisition, Writing – original draft. **Domenico Prisco:** Conceptualization, Supervision, Project administration, Funding acquisition, Writing – original draft. **Annarosa Arcangeli:** Conceptualization, Supervision, Project administration, Funding acquisition, Writing – original draft, Writing – review & editing.

## Declaration of Competing Interest

The authors declare that they have no known competing financial interests or personal relationships which have or could be perceived to have influenced the work reported in this article.

## Data Availability

Data were submitted to GEO under accession number GSE145191.
